# Liquid Biopsy in Cancer: The Importance of Integrating Bioinformatics Approaches Towards the Development of a Personalized Molecular Profile

**DOI:** 10.3390/cancers18010140

**Published:** 2025-12-31

**Authors:** Areti Strati, Kostas A. Papavassiliou, Athanasios G. Papavassiliou

**Affiliations:** 1 Department of Biological Chemistry, Medical School, National and Kapodistrian University of Athens, 11527 Athens, Greece; 2 First University Department of Respiratory Medicine, ‘Sotiria’ Chest Hospital, Medical School, National and Kapodistrian University of Athens, 11527 Athens, Greece; konpapav@med.uoa.gr

Liquid biopsy is now a valuable complementary tool that oncologists use to obtain a more complete picture of their patients’ condition in real time [1]. This enables them to monitor patients or intervene as needed to further investigate or adjust treatment [2]. In lung cancer, liquid biopsy has been used in numerous studies analyzing various biomarkers in blood, bronchoalveolar lavage (BAL), cerebrospinal fluid (CSF), and other fluid samples [3]. However, this information is complex and can be simplified with bioinformatics tools [4]. Starting from molecular/minimal residual disease (MRD) analysis, research can advance to multi-omic, fragmentomic, and transcriptomic analyses, making bioinformatics essential for translating liquid biopsy data into clinically actionable biomarkers for early detection, prognosis, and therapeutic decision-making in precision oncology [5].

Bioinformatics has been used to identify the molecular mechanisms underpinning relevant networks, pathways, and potential biomarkers related to *tumor metastasis* [6]. The TRAcking Cancer Evolution through therapy/Rx (TRACERx) program exemplifies integrative bioinformatics in large-scale longitudinal studies by leveraging high-dimensional genomic data to track early metastatic spread and subclonal dynamics [7]. The bioinformatic tool ECLIPSE is specifically designed for non-invasive tracking of subclonal architecture at low circulating tumor DNA (ctDNA) levels. ECLIPSE identified patients with polyclonal metastatic dissemination, which was associated with poor clinical outcomes. Complementary analyses have validated clonal expression-based biomarkers, such as Outcome Risk Associated Clonal Lung Expression (ORACLE), which correlate with survival and predict chemotherapy sensitivity, underlining the clinical relevance of integrating evolutionary and transcriptional information across solid tumors [8].

Recent advances in liquid biopsy and computational modeling have enabled the development of dynamic biomarkers that outperform traditional static clinicopathologic features. Patient-specific pROgnostic and Potential tHErapeutic marker Tracking (PROPHET) uses deep sequencing of patient-specific somatic variants to achieve ultra-sensitive MRD detection and earlier relapse prediction than fixed-panel assays [9]. A novel serial quantitative tumor fraction algorithm, xM, was applied to data from the Tempus de-identified clinical genomic database, which includes 14 solid cancer types in patients who received longitudinal liquid-based next-generation sequencing (NGS). The results show that xM can be used clinically to evaluate immune checkpoint inhibitor (ICI) therapy efficacy [10]. Similarly, the integration of immune profiling and pre-treatment ctDNA status could predict which patients will achieve durable clinical benefit (DCB) from immune checkpoint inhibition [11].

A novel machine-learning approach capable of exploring the full landscape of mutations and combinations could identify new predictive biomarkers for chemoimmunotherapy, underscoring the importance of modeling combinatorial mutation patterns rather than single-gene effects [12]. In parallel, integrative frameworks that combine longitudinal ctDNA kinetics with peripheral immune biomarkers such as neutrophil-to-lymphocyte ratio or eosinophil dynamics, capture evolving tumor–immune interactions that correlate with clinical outcomes following immune checkpoint blockade across multiple cancer types [13].

Multi-level classification frameworks that integrate cell-free DNA (cfDNA)/ctDNA genomic features with circulating protein biomarkers illustrate how staged binary classifiers, ensemble learning, and consensus feature selection can achieve high diagnostic accuracy across diverse malignancies, including small-cell lung cancer (SCLC) and non-small-cell lung cancer (NSCLC), colorectal cancer (CRC), breast cancer, and gastrointestinal (GI) cancers [14]. Another major advancement has been the use of non-mutational cfDNA signals and transcriptomic diversity to address sensitivity and cost limitations [15]. Complementary transcriptome-based liquid biopsy approaches including circulating microRNAs (miRNAs), exosomal RNAs (exRNAs) [16], and platelet-derived mRNA and circular RNA (circRNA) signatures [17] show that computational feature extraction and combinatorial modeling can reveal cancer-specific signals missed by single-analyte assays. Population-scale analyses of longitudinal circulating RNA profiles further suggest that molecular dysregulation may precede clinical cancer diagnosis by years or even decades, highlighting the potential for ultra-early detection through large-scale bioinformatic data mining [18].

Advanced feature selection, class imbalance correction, and ensemble modeling improve predictive performance in high-dimensional proteomic and transcriptomic datasets [19], while decision-support algorithms that integrate ctDNA with circulating protein markers demonstrate clear clinical utility when tissue biopsies are inconclusive or infeasible [20]. Thus, a key issue is that bioinformatics contributes not only predictive accuracy but also interpretability and translational relevance. Additionally, tumor-educated platelet (TEP)-derived spliced RNAs selected by optimized machine learning algorithms may serve as biomarkers for minimally invasive clinical blood tests [21].

Recent progress in liquid biopsy-based NGS for NSCLC highlights a critical transition from variant detection toward quantitative and reliability-aware interpretation of molecular data. According to Serna-Blasco et al. [22], the R-score represents an important methodological contribution to this shift, addressing a long-standing gap in objective quality metrics for NGS variant calls in plasma [22]. By integrating variant allele fraction (VAF) and the Median of the Absolute values of all Pairwise Differences (MAPD) into a single parameter that correlates strongly with positive percent agreement across platforms, the R-score provides a practical, data-driven framework for reducing false-positives and false-negative variant calls in ctDNA analysis. This concept aligns well with broader efforts in the field to improve analytical robustness, such as ultra-deep sequencing strategies for early detection [23], algorithmic integration of clinical and molecular variables [24], and emerging approaches that combine DNA and RNA profiling to maximize actionable insight from limited material [25].

In conclusion, the rapid evolution of liquid biopsy technologies, along with advances in bioinformatics and machine learning, has transformed cancer research and clinical management across various tumor types. These developments illustrate a paradigm shift in liquid biopsy-based precision oncology, where bioinformatics-driven integration, modeling, and quality control are as critical as sequencing technology itself. By linking molecular dynamics with clinical and immune context, these approaches move the field beyond static, single-marker assays toward scalable, reproducible, and clinically deployable decision-support systems applicable across a broad spectrum of solid cancer types.
